# A high-throughput screening system for barley/powdery mildew interactions based on automated analysis of light micrographs

**DOI:** 10.1186/1471-2229-8-6

**Published:** 2008-01-23

**Authors:** Alexander Ihlow, Patrick Schweizer, Udo Seiffert

**Affiliations:** 1 Pattern Recognition Group, IPK Gatersleben, Corrensstr. 3, D-06466 Gatersleben, Germany; 2 Transcriptome Analysis Group, IPK Gatersleben, Corrensstr. 3, D-06466 Gatersleben, Germany

## Abstract

**Background:**

To find candidate genes that potentially influence the susceptibility or resistance of crop plants to powdery mildew fungi, an assay system based on transient-induced gene silencing (TIGS) as well as transient over-expression in single epidermal cells of barley has been developed. However, this system relies on quantitative microscopic analysis of the barley/powdery mildew interaction and will only become a high-throughput tool of phenomics upon automation of the most time-consuming steps.

**Results:**

We have developed a high-throughput screening system based on a motorized microscope which evaluates the specimens fully automatically. A large-scale double-blind verification of the system showed an excellent agreement of manual and automated analysis and proved the system to work dependably. Furthermore, in a series of bombardment experiments an RNAi construct targeting the *Mlo *gene was included, which is expected to phenocopy resistance mediated by recessive loss-of-function alleles such as *mlo5*. In most cases, the automated analysis system recorded a shift towards resistance upon RNAi of *Mlo*, thus providing proof of concept for its usefulness in detecting gene-target effects.

**Conclusion:**

Besides saving labor and enabling a screening of thousands of candidate genes, this system offers continuous operation of expensive laboratory equipment and provides a less subjective analysis as well as a complete and enduring documentation of the experimental raw data in terms of digital images. In general, it proves the concept of enabling available microscope hardware to handle challenging screening tasks fully automatically.

## Background

Recent molecular methods have paved the way for a number of new experimental approaches in life science which were not available several years ago. As a matter of fact, these new techniques exceed the capacity of well-established manual or scantily automated analysis by far. Automated high-throughput analysis techniques not only solve this problem – they generally introduce a less subjective, more reproducible, and potentially more accurate data processing. However, competing with intuitive and trainable human skills, even though only for a rather specific problem, often turns out to be a difficult task.

This paper introduces a fully automated high-throughput screening system which has been developed for supporting a functional genomics approach in the field of plant-pathogen interactions.

**Figure 1:**
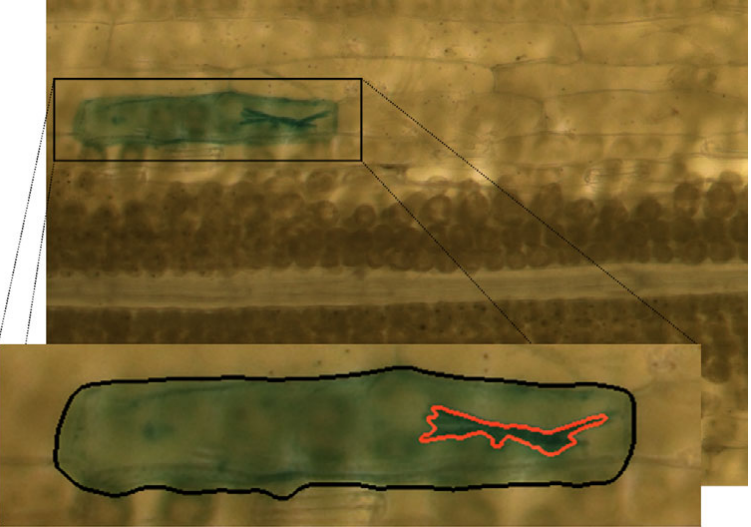
**Light micrograph of prepared barley leaf epidermis**. Epidermal cell tissue with a well stained, genetically transformed cell and a salient haustorium. In the enlarged region of interest, the cell boundary and the haustorium are marked in black and red, respectively.

In order to directly assess gene function in barley and wheat suffering from biotic stress caused by the powdery mildew fungus *Blumeria graminis*, a transient assay system based on bombarded leaf epidermis was developed and proved to be useful [1-4]. This system, which can be used for transient overexpression of genes as well as for transient-induced gene silencing (TIGS), has recently been further developed using GATEWAY technology in order to enhance throughput [5]. In the experiments, young barley leaves are bombarded with DNA-coated gold particles which confer transient expression of desired genes. For analysis purposes, the beta-glucuronidase (GUS) reporter gene is co-expressed in cells that were hit by the particle bombardment. This stains the genetically transformed cells greenish blue and allows their identification by bright field microscopy. In the evaluation of the experiments, haustoria of the powdery mildew have to be detected inside these stained, genetically transformed cells, as they indicate a successful penetration by the fungus. A cutout of a typical micrograph, containing a well stained, transformed cell with one haustorium is depicted in Figure 1. By evaluating several hundred transformed cells per test gene, the susceptibility of the cells to the fungus is assessed in terms of the susceptibility index

$$SI=\frac{\text{number of infected transformed cells}}{\text{total number of transformed cells}}.$$

A significant increase or decrease of this index indicates a relation of the test gene to the plant's defense mechanism.

Manual screening has been done for a large number of experiments and this proved to be a tedious and very time-consuming mission. The desired screening of thousands of candidate genes would require many person years without automation. Relegating this task to a fully automated high-throughput screening system offers a number of advantages: Besides saving labor, the subjective component of the human observer is replaced by deterministic image analysis algorithms. Due to the autonomous operation, continuous activity (24/7) becomes possible, leading to a higher utilization degree of expensive laboratory equipment. Last but not least, the intrinsic storage of the experimental raw data as digital images provides a complete and enduring documentation of the experiments for further reference.

## Results and discussion

### Screening pipeline

**Figure 2:**
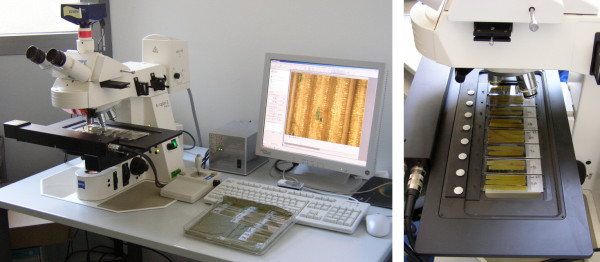
**Microscope hardware**. The hardware basis of the developed high-throughput screening system is a motorized, computer-operated light-optical microscope (*left*). The xy-table can carry up to 8 slides (*right*).

The hardware basis is a motorized, computer-operated light-optical microscope (Axioplan 2 imaging, Carl Zeiss, Germany), including an xy-stage for up to eight slides and a CCD camera (AxioCam HRc, Carl Zeiss, Germany). Figure 2 depicts the hardware. The microscope is controlled by the vendor's software AxioVision. Automation is achieved by a script program which operates AxioVision via its optionally available Visual Basic for Applications (VBA) interface. This script program also provides a graphical user interface where the experimenter parametrizes and starts the screening. It cooperates with a set of specifically developed stand-alone image analysis programs, which subsequently evaluate the images directly after acquisition. The resulting screening pipeline consists of two main domains: image acquisition and image analysis. First, let us focus on important aspects of the image acquisition and their consequences for the design of the system.

**Figure 3:**
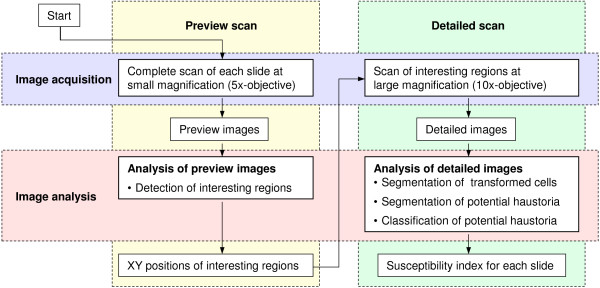
**Screening pipeline**. After inserting the slides onto the xy-stage, the screening is started in the control program. The final output are the susceptibility indices for each slide in terms of the number of infected transformed cells over the total number of transformed cells according to Equation (1).

To realize a fully automated specimen analysis, interesting regions on the slide must first be identified, before detailed images of these regions can be generated. A naive complete high-resolution scanning is out of the question due to the tight time constraints of a high-throughput system. Therefore, the screening is divided into two main temporal passes, namely a preview scan and a subsequent detailed scan. During the preview scan, an overview image of each slide is generated by assembling together coarse resolution images (432 × 342 pixels) of low magnification (5× objective). To cover the entire slide, 15 horizontal times 38 vertical steps are necessary, leading to 570 subimages per slide. After the positions of transformed cells are identified, the detailed scan starts. Subsequently, the microscope changes to the 10× objective and acquires high-resolution images (1300 × 1030 pixels) of each requested position. To overcome the limited depth of field, images of several foci are necessary. Five focal layers of 7 *μ*m distance proved to be sufficient to provide a sharp reproduction of all details. The described pipeline is illustrated in Figure 3 as a flow chart.

### Time constraints

From the user's perspective, an important aspect is the expentiture of time for a screening. Here the limiting factor is the image acquisition, which is dependent on the microscope hardware, whereas the subsequent image analysis proves to be noncritical on current computer hardware. Table 1 summarizes the time required for the individual image acquisition steps. Note that these numbers are expected to decrease significantly on future microscope hardware. With our current configuration we are able to load the microscope three times a day (7 am, about noon, and about 4 pm) each with eight slides, whereby the last run completes in the evening.

**Table 1:** Expenditure of time for image acquisition

Action	1st pass: preview scan (5×-objective, 432 × 342 pixels)	2nd pass: detailed scan (10×-objective, 1300 × 1030 pixels)
stage positioning	≈ 0.5 s	≈ 1 s
auto focus	negligible (once per slide)	≈ 3 s
single shot	≈ 0.7 s	≈ 1.5 s
several focal layers	--	5 layers per position
time expenditure	≈ **10 min **per slide (scanning 15 × 38 = 570 positions)	≈ **20 min **per slide (scanning ≈ 100 positions*)

*A number of 100 positions is an average, depending on the actual quantity of transformed cells on the slide.

### System output and intermediate results

**Figure 4:**
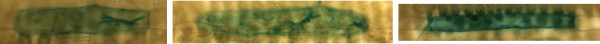
**Typical examples of transformed cells**. Both the left and the centered cell contain one haustorium of the powdery mildew fungus.

**Figure 5:**
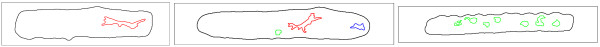
**Automatically generated sketches of the cell images of Figure 4**. The cell boundary appears in black. Objects classified as haustoria are marked in red, other objects in green, ambiguous objects in blue.

After processing the detailed images, the system primarily provides the susceptibility indices according to Equation (1) for each scanned slide in terms of the number of infected transformed cells and the total number of transformed cells. The detailed description of necessary image processing and pattern recognition algorithms might be of limited interest for potential users, but fundamental for researchers and developers who need to completely understand the system. Therefore, at this point we give an illustrative overview of the image analysis and refer the reader to the method section for a detailed disquisition. Figure 4 exemplarily shows three typical stained cells, in which both the left and the centered cell contain a haustorium. Figure 5 illustrates the results of the automated image analysis, consisting of three main steps: First, the cell segmentation finds transformed cells in the image and provides the exact cell boundary (displayed in black color). Afterwards, the haustoria segmentation detects potential haustoria (displayed inside the cell by their contours). As a last step, each segmented object is validated by the haustoria classification as to whether it is truly a haustorium or a false positive object. In the sketch, this is illustrated by the object's colors: Objects classified as haustoria are marked in red, other objects appear green. There are rare cases, in which even an experienced expert is in doubt about the classification of questionable objects. The same holds for the system: The middle cell contains an object (displayed as a blue contour) which can neither be classified as haustorium nor as other object. As it would be done in case of a manual screening, cells without a reliable decision will be excluded from the calculation of the susceptibility index.

### System validation

Benchmarking the system gives evidence about its reliability. One problem, the rather variable staining intensity of the transformed cells, can be dealt with by sophisticated image processing algorithms to detect these properly. But the generally low contrast of haustoria as well as the occurrence of salient discolorations in the stained cells preclude a naive haustoria detection solely on weak color differences to the staining. For a reliable classification, we applied machine-learning techniques which are trained on a reference data set previously labeled by an expert human observer. Based on this manually labeled data set, we investigated a classification accuracy of 95 ± 1%.

**Figure 6:**
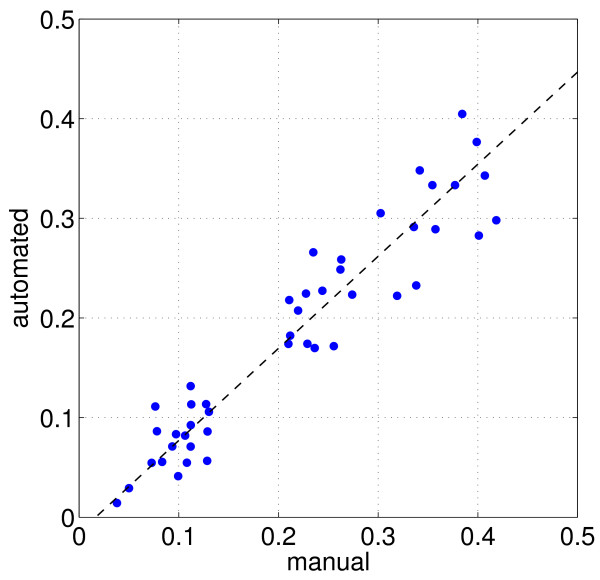
**Correlation between manual and automated analysis**. In this evaluation, 45 experiments (each consisting of two microscopic slides) were analyzed by a human expert as well as by the automated system. Measured quantity is the susceptibility index *SI*, which is given by Equation (1).

Ultimately, the screening system has to meet the standards of meticulous manual evaluation. As an exhaustive verification, we performed a large-scale double-blind test: A number of 45 experiments, each consisting of two microscopic slides with hundreds of transformed cells, were manually screened by one human expert by counting both the number of infected transformed cells (which contain at least one haustorium) and the total number of transformed cells. According to Equation (1), this gives a certain susceptibility index. Additionally, the slides were screened by the automated system and the susceptibility index was calculated using the automatically derived classification results. Plotting the susceptibility indices for each experiment against each other, Figure 6 shows the congruence in the results of both analysis methods. Clearly, we do not know the true susceptibility indices since both the system and the human observer are not infallible and errors may occur from both parts. However, the strong accordance of automated and human analysis with a correlation coefficient *R *> 0.9 indicates that the system works dependably.

From the biological point of view, only large changes of the susceptibility index are significant and must be reliably detected by the system. Evidently, this goal is reached in practice.

### Screening for gene discovery

**Figure 7:**
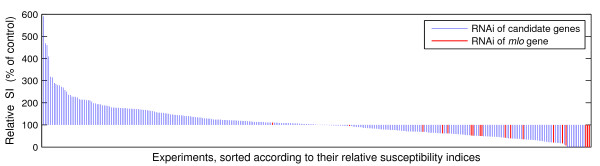
**Results of an automated TIGS screening**. The screening comprised ≈ 300 defense-related candidate genes (displayed as blue bars). As a positive control for RNAi efficiency, a construct targeting the *Mlo *gene was also included (displayed as red bars). As to be expected, the relative susceptibility indices that resulted from the RNAi construct targeting *Mlo *were clustered at the more resistant side of the spectrum.

The automated analysis system was used for a TIGS screening of more than 300 defense-related candidate genes previously found to be up-regulated in pathogen-attacked barley epidermis. As a positive control for RNAi efficiency, a construct targeting the *Mlo *gene was also included, which is expected to phenocopy resistance mediated by recessive loss-of-function alleles such as *mlo5*. As shown in Figure 7, TIGS of the different barley candidate genes resulted in a broad range of relative susceptibility indices, and many within the "extrema" groups could be reproduced in four additional, independent repetitions (data to be published elsewhere). Importantly, the relative susceptibility indices that resulted from the RNAi construct targeting *Mlo *were clustered at the more resistant side of the spectrum, as reported previously for manually scored TIGS experiments using the same construct [5]. This provided proof of concept for a usefulness of the automated system for de-novo discovery of genes affecting the barley/powdery mildew interaction.

## Conclusion

The described high-throughput screening system enables a large-scale analysis of candidate genes regarding the resistance of crop plants against the powdery mildew fungus by automating very time-consuming screening tasks. Proved to work dependably and at operational stage now, it provides a novel tool of medium- to high-throughput phenomics in the crop plant barley allowing researchers to address gene function in host- or nonhost interactions for resistance. A single experimenter is expected to test up to 100 candidate genes per person month, which is approximately two orders of magnitude higher than whole-plant approaches in barley such as stable transgenic plants or TILLING mutants. Currently, the system is established in a number of projects at our research institute as well as at international cooperating partners.

As a general conclusion, the developed solution can be understood as a proof of concept of how to extend already available microscope hardware to handle challenging screening tasks fully automatically by bringing together research and development both from the fields of biology and engineering. Of course, this concept is neither limited to the described application nor to the currently used microscope hardware. In the future, the system will be adapted to further challenges and we will focus also on screening problems incorporating fluorescence microscopy. This paper should encourage other researchers to tackle analogous screening tasks in a similar way.

## Methods

**Figure 8:**
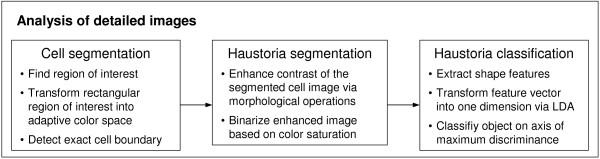
**Image analysis pipeline**. This figure is a more detailed description of the corresponding box of Figure 3.

Having explained the general system concept, its time constraints, and reliability, we will now discuss in detail the image analysis. This information is essential to completely understand the functioning of the system in assessing the infection status of transformed cells by haustoria. The image analysis pipeline is illustrated in Figure 8 and will be presented here in detail. Firstly, the specimen preparation is considered.

### TIGS screening

The screening was carried out in seven-day-old susceptible barley plants of cv. Golden Promise, as described in [5]. Briefly, leaf segments were bombarded by gold particles that had been coated with a mixture of pUbiGUS (reporter plasmid) and pIPKTA30 Target (RNAi plasmid) using a PDS 1000/He system (Bio-Rad, Munich, Germany). Three days after the bombardment, leaf segments were inoculated with *Blumeria graminis *f. sp. *hordei *and incubated for another 40 h, followed by staining of transformed cells for beta-glucuronidase (GUS) activity and microscopy.

### Cell segmentation

**Figure 9:**
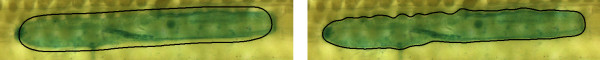
**Cell segmentation via an active contour**. The approximate cell boundary found by edge detection (*left*) is iteratively refined by moving the contour towards the true edge of the stained cell (*right*).

Due to the performed staining (cf. Figures 1 and 4), hue is an apparent feature to discriminate between the transformed cells and the surrounding epidermal tissue. To cope with the large variability of the staining intensity and to detect also regions with very weak dyestuff expression, we use an edge detection approach [6,7]. As an intermediate result, the stained cells are marked by a closed contour. To refine this recognition and to properly detect the exact cell boundary, more sophisticated techniques are applied afterwards: First, the rectangular region of interest containing the stained cell is transformed into an adaptive color space [8], incorporating the Expectation Maximization (EM) algorithm [9,10] for parameter estimation. Compared to hue, this adaptive color space provides a better discriminatory power between the staining and the color of the surrounding tissue. It is applied to the previously detected regions of interest only, because of its high computational cost. Subsequently, based on the adaptive color space the estimated approximate cell boundary contour is moved towards the nearby true edge of the stained cell using an active contour model. Following the classical formulation according to Kass *et al*. [11], we implemented a new, computationally more efficient numerical solution scheme [12]. The result is illustrated in Figure 9: Initialized with the rough contour (depicted in the left image), the active contour model finally provides the accurate cell boundary (depicted in the right image). This high effort to gain exactly the cell boundary is justified due to salient discolorations of the cell wall, which occasionally show the same color features as potential haustoria. They would interfere during the next processing step and must be removed in advance.

### Haustoria segmentation

Having isolated the stained, genetically transformed cells, potential haustoria must be detected therein. Generally, both haustoria and other salient objects exhibit a slightly more saturated color than the remaining cell area. Exploiting this feature, we perform the following heuristic: First, a contrast enhancement via morphological top-hats [13] is applied on the color image of the cell cutout. Since morphological top-hats operate *contrarily *by extracting objects which *cannot *contain the structuring element, a rectangular or disk-shaped structuring element being somewhat larger than a haustorium is appropriate. (Actually, we use a disk-shaped structured element of 37 pixels diameter.) This leads to a content-adaptive contrast enhancement, increasing the saliency of potential haustoria regions while preserving the saliency of the remaining cell area. Second, the color saturation of the enhanced image cutout is taken as feature image for the segmentation. Salient regions are extracted by a region growing segmentation starting from seeds which exceed a certain high threshold until falling below a second, low threshold. This is efficiently realized by using a binary morphological reconstruction method [14]. We describe this haustoria segmentation process in detail in [15].

At this stage, where the stained cells have been segmented and potential objects that might be haustoria have been marked, the image processing part is completed. Looking back at Figures 4 and 5 we now have obtained a sketch of the color images, representing the objects of interest. As a last milestone, the identified potential haustoria regions must be classified into true haustoria and false positive objects. In Figure 5 this is illustrated by the color, where haustoria are marked in red.

### Haustoria classification

To transfer the expert knowledge, enabling the human observer to detect haustoria, to the machine, distinctive features of the considered objects are extracted and fed into an appropriate classifier. In general, the composition of an adequate feature vector is the most important step to obtain a good classification performance. Furthermore, the selected features have to be considered in relation to the subsequent classification technique. In [16] we tested several feature combinations on sophisticated classifiers and drew the conclusion that a classification accuracy of more than 90% is possible.

Due to an improved feature preprocessing we recently simplified the solution by enabling the use of a linear classifier. Confinement to an uncomplex classifier has the advantage of being independent of further specifications such as neural network topologies, training algorithms, learning rates, or other parameters. In addition, there is the least risk of overfitting. To reach this aim, the feature vector is adapted by nonlinear transformations in advance. As an example, consider incorporating the object's perimeter *P *and area *A*. For a given object, both features are related nonlinearly by *A *~ *P*^2^. Hence, it can help a linear classifier to adapt to this feature vector when the object area is incorporated as $\sqrt{A}$, as the former nonlinear correlation of both features is thus linearized. Such a nonlinear preprocessing can also be done for more sophisticated features.

In order to train a classifier onto the extracted features, a representative data set is needed which contains samples of virtually all possible cases both of the classes *"haustorium" *and *"other object"*. Therefore, we manually annotated a large set of digital images containing transformed cells with and without haustoria and stopped when the data set comprised 500 objects for each class.

#### Shape features for haustoria recognition

In order to complement the color features which have already been exploited during the segmentation, we must now focus on the object's shape. For haustoria recognition, the features must reflect a class of specifically shaped objects, consisting of a body with "fingers". Therefore, beside basic shape descriptors such as the object's area, its contour length, or principal axes, we incorporate two sophisticated approaches: moment invariants [17,18] and Fourier descriptors [19].

As appropriate moment invariants, we use the set *ψ*_1_...*ψ*_11 _introduced by Flusser [18]. To enable their use with a linear classifier, the following transformation is applied:

$$\begin{array}{ccc} {\tilde{\psi }}_{i}=\sqrt{\left|\psi_{i}\right|\xi_{i}} & \text{with} & \xi_{1...11}=\{2,6,8,8,12,12,4,10,10,16,16\}. \end{array}$$

Moment invariants can be derived both from a gray-level image and a binarized image. We incorporate the invariants ${\tilde{\psi }}_{1}...{\tilde{\psi }}_{11}$ of both the binary image and the color saturation image, leading to 22 features.

In addition to region-based features, we further use a set of contour-based features in terms of Cartesian Fourier descriptors [19]. Therefore, the *x*- and *y*-coordinates of the sampled contour of length *N *are taken as complex numbers *z*_*n *_= *x*_*n *_+ j *y*_*n *_(*n *= 0...*N *-1) and the periodic sequence is represented by its spectral coefficients ${\hat{Z}}_{\nu }=\frac{1}{N}\sum_{n=0}^{N-1}z_{n}{\text{e}}^{-\text{j}2\pi \nu n/N}$, also known as Fourier descriptors. The set

$$\left[\frac{{\left|{\hat{Z}}_{-n}\right|}^{2}}{{\left|{\hat{Z}}_{1}\right|}^{2}}\frac{{\left|{\hat{Z}}_{-(n-1)}\right|}^{2}}{{\left|{\hat{Z}}_{1}\right|}^{2}}\cdots \frac{{\left|{\hat{Z}}_{-1}\right|}^{2}}{{\left|{\hat{Z}}_{1}\right|}^{2}}\frac{{\left|{\hat{Z}}_{2}\right|}^{2}}{{\left|{\hat{Z}}_{1}\right|}^{2}}\cdots \frac{{\left|{\hat{Z}}_{n}\right|}^{2}}{{\left|{\hat{Z}}_{1}\right|}^{2}}\right]$$

is invariant to shifting due to omitting the descriptor ${\hat{Z}}_{0}$ and invariant to scaling due to normalization by ${\hat{Z}}_{1}$. Furthermore it is invariant to rotation due to using the absolute value of the complex Fourier coefficients. In fact, this power spectrum describes the contour in terms of its autocorrelation features – its linear self-similarity. Since the spectrum decays rapidly towards higher frequencies we use the following equalization and introduce the transformed Fourier descriptors

$${\tilde{\hat{Z}}}_{\nu }={\left|\frac{{\hat{Z}}_{\nu }}{{\hat{Z}}_{1}}\right|}^{\frac{1}{\left|\nu \right|}}.$$

In the feature vector we use ${\tilde{\hat{Z}}}_{-14}\ldots {\tilde{\hat{Z}}}_{14}$, which leads to 27 features due to omitting ${\hat{Z}}_{0}$ and ${\hat{Z}}_{1}$. So far, composed of moment invariants and Fourier descriptors, the feature set is scale invariant, i.e., the information about the object's size is not contained. This changes through the incorporation of the square root of object area $\tilde{A}=\sqrt{A}$, the object perimeter *P*, and the major and minor axis length *a *and *b*, respectively. Additionally, we consider the normalized multiscale bending energy (NMBE) Ψ [20,21] in terms of $\tilde{\Psi }=\sqrt{1/\Psi }$ and the mean color saturation *S *as features. Together with the 22 moment invariants and the 27 Fourier descriptors this finally leads to a set of 55 features.

After normalizing the feature vector to zero mean and unit variance by applying the standard score (also called z-score or normal score) transformation [22], it is ready for the actual classification.

#### Classification

**Figure 10:**
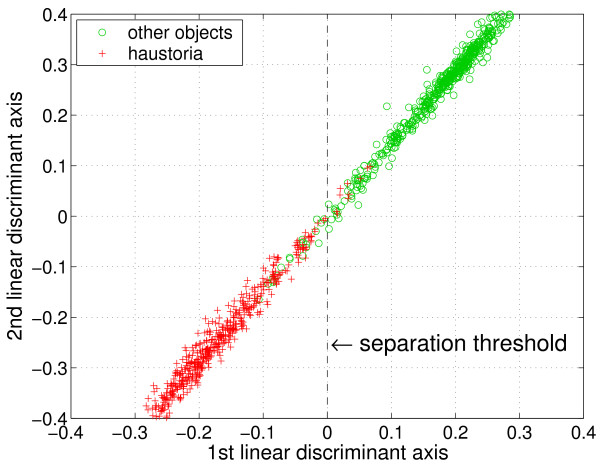
**Object classification by linear discriminant analysis**. The 55-dimensional feature space, in which both haustoria and other objects are described, is reduced to two dimensions. The linear discriminant analysis (LDA) projects the data onto axes of maximum discriminatory power. Both dimensions are highly correlated, so that considering multiple linear discriminant axes yields no benefit. Hence, the 1st axis is sufficient for classification.

In [16] we have, inter alia, used the linear discrimant analysis (LDA) [22] as a feature reduction technique. The LDA linearly weights the input features in such a way that the output dimension exhibits maximum discriminatory power. Inititally developed by Fisher [23], it is extended in [24] to multiple output dimensions by building an orthonormal system yielding maximum discriminatory power in each dimension. The resulting transformed, reduced feature set can be applied to any classification algorithm. In case the classes are linearly separable, the LDA itself is sufficient already for classification. Due to the previously applied nonlinear feature transformations we have successfully generated this ideal configuration: In Figure 10 the projection of all 55 features onto two dimensions via LDA is depicted. Both dimensions show a high correlation. Incorporating a third discriminant axis would show a similar highly-correlated scenario in 3-D. As a result, classification based on the axis of maximum discriminatory power is sufficient, i.e., simply a separation threshold needs to be applied to the 1st discriminant axis (cf. Figure 10).

For estimating the expected classification performance on unknown data, the representative data set needs to be partitioned into a training subset and a disjoint test subset [25]. We randomly partitioned the data set and used one half for training the classifier and the other half for testing. To obtain a stable informational value, this partitioning, training, and testing was performed in terms of 500 different realizations. As a result, we obtained a classification accuracy of 95 ± 1%.

At the end of this analysis pipeline, all necessary information is available to distinguish infected cells from uninfected ones. Cells are considered as infected if there is at least one object classified as a haustorium.

## Authors' contributions

AI developed the screening system. PS coordinated and mentored the biological part of the development. US coordinated and mentored the engineering part of the development. All authors read and approved the final manuscript.
